# Prothrombin Gene G20210A Variant in Angiographically Documented Patients with Coronary Artery Stenosis

**Published:** 2019-10

**Authors:** Leyla Pourgholi, Hamidreza Goodarzynejad, Shayan Ziaee, Elmira Zare, Arash Jalali, Mohammadali Boroumand

**Affiliations:** 1 *Department of Molecular Pathology, Tehran Heart Center, Tehran University of Medical Sciences, Tehran, Iran.*; 2 *Department of Cardiac Research, Tehran Heart Center, Tehran University of Medical Sciences, Tehran, Iran.*

**Keywords:** *Coronary artery disease*, *Prothrombin*, *Coronary angiography*, *Single-nucleotide polymorphism*

## Abstract

**Background: **Studies on the association between the prothrombin G20210A variant and coronary artery disease (CAD) risk are inconclusive. This study aimed to investigate the possible association between the G20210A variant in the prothrombin gene and documented CAD and its severity.

**Methods: **This study enrolled 1460 patients who were consecutively admitted for elective coronary angiography. Via the standard angiographic techniques, coronary angiographies were done and the presence and severity of CAD were determined through the clinical vessel score and the Gensini score. Prothrombin G20210A genotypes were identified using PCR-RFLP.

**Results: ** This cross-sectional study was performed on 953 men and 507 women at a mean age of 58.21±10.33 years. The median and the interquartile range for the Gensini score were not statistically significantly different between the wild (GG) and mutant (AA+GA) genotypes (P=0.440). The association between the G20210A polymorphism and the severity of CAD with respect to the vessel score also showed no significant linear trend of higher numbers of diseased vessels (P= 0.765 for the Mantel–Haenszel test of linear trend) in the AA+GA genotype as compared with the GG genotype.

**Conclusion:** Our data failed to confirm the hypothesis that the G20210A variant mutation may be a significant determinant of CAD risk or its severity.

## Introduction

Prothrombin (coagulation factor II), the precursor of the serine protease thrombin, is a key procoagulant enzyme acting through platelet activation and forming fibrin and factors Va, VIIIa, and XIIIa. It subsequently acts as an anticoagulant through activating circulating protein C. The 21-kilobase (kb) gene that codes the protein is mapped to chromosome 11 (11p11-q12).^   1 ^ The G20210A single-nucleotide polymorphism (SNP) of the factor II gene is due to a substitution of adenine for guanine at nucleotide 20210, located in the 3’-untranslated region, and is associated with elevated serum prothrombin levels and an increase in the risk of venous thrombosis by approximately threefold.^   2 ^ However, the role of this variant in a higher risk for thrombosis localized to the arterial circulatory system still remains controversial.^   3^

Coronary artery disease (CAD), a major health problem and the leading cause of death in the industrialized countries, is a complex multifactorial disorder influenced by environmental factors and complex patterns of inheritance.^   4 ^ Studies on the association between the prothrombin G20210A variant and CAD often have yielded conflicting results.^5^^-^^11^  In a comprehensive meta-analysis of 15 041 cases and 21 507 controls, the G20210A variant of factor II was related to a higher CAD risk in Europeans, but no statistical differences were found between Americans and Asians.^   3 ^ Repeated studies in different settings and populations are required to assess whether this variant can influence the risk of cardiovascular disease. The aim of the current study was, therefore, to investigate whether the presence of the G20210A variant in the prothrombin gene is related to the presence of documented CAD and its severity in a relatively large sample of Iranian patients who underwent coronary angiography.

## Methods

This cross-sectional study recruited 1460 patients who were consecutively admitted for elective coronary angiography at our institution. Patients with angiographic evidence of atherosclerosis (≥ 50% luminal stenosis in the epicardial coronary tree) were categorized as having CAD (CAD group, n=995). Patients with no luminal stenosis (n=286) or patients with less than 50% luminal stenosis (n=179) on coronary angiography were categorized as controls (non-CAD group). All the procedures performed were in accordance with the ethical standards of the institutional and/or national research committee and with the 1964 Helsinki Declaration and its later amendments. The local ethics committee approved the study protocol, and written informed consent was obtained from all individual participants included in the study.

Via standard angiographic techniques, coronary angiographies were performed and the presence and severity of CAD were determined using the clinical vessel score. The angiograms were classified as revealing either no coronary lesions (absent); no coronary lesions with more than 50% luminal stenosis (minimal); or 1 (mild), 2 (moderate), or 3 (severe) major epicardial coronary arteries with more than 50% luminal obstructions. Patients with less than 50% luminal stenosis were categorized as having minimal CAD. The severity of CAD was also determined using the Gensini score, a semiquantitative scoring system which has been previously described.^   12 ^ All the angiograms were interpreted by 2 cardiologists, blinded to the study results. The definitions of the analyzed risk factors of CAD including age, male sex, hyperlipidemia, hypertension, cigarette smoking, and diabetes have been previously reported.^   13^

Peripheral venous blood specimens were collected from the patients’ antecubital vein after they had 12 hours’ overnight fasting. Genomic DNA was extracted from leukocytes via the standard salting out procedure using the buffy coat of EDTA specimens.^   15 ^

For the genotype analysis, polymerase chain reaction–based restriction fragment length polymorphism (PCR-RFLP) was applied. The oligonucleotide primers used to determine the prothrombin polymorphism included forward primer, 5' - TCT AGA AAC AGT TGC CTG GC - 3'; and reverse primer, 5' - TGC CCA TGA ATA GCA CTG GGA GCA TTG AGG AT - 3'. (The mismatched nucleotide is presented as an underlined letter.) A typical PCR comprised template DNA (100 ng), each of the forward and reverse primers (0.2 μM), the dNTP mixture (180 μM of the final concentration for each dNTP), MgCl2 (1.5 mM), and Taq DNA polymerase (1.25 U) in a total volume of 50 μL. The PCR conditions consisted of initial denaturation at 95^°^C for 10 minutes, 35 cycles of denaturation at 95^°^C for 30 seconds, primer annealing at 56^°^C for 30 seconds, extension at 72^°^C for 20 seconds, and final extension at 72^°^C for 5 minutes. The amplified PCR products were then digested with the Mbo I restriction enzyme for genotypic analysis. The distinction between the heterozygotes, homozygotes, and noncarriers of these mutations was assessed by agarose gel electrophoresis 3%, stained with ethidium bromide.

The genotypes were termed “AA”, “GA”, or “GG”, where the A allele codes for the presence of the risk allele. The mutated homozygous variant AA produced 2 fragments of 207 bp and 145 bp, whereas the heterozygote AG produced 4 fragments of 207 bp, 145 bp, 114 bp, and 31 bp. The wild-type GG produced 3 fragments of 207 bp, 114 bp, and 31 bp.

All the statistical analyses were performed using the SPSS software, version 18.0, for Windows (SPSS Inc., Chicago, IL). The continuous variables were presented as the mean±standard deviations ^   16 ^ or the median with interquartile ranges (IQRs) and were compared between the CAD and non-CAD groups and also between the prothrombin genotypes using the Student *t* test or the Mann–Whitney *U* test. Since the distributions of the Gensini scores were skewed and could not be normalized by several transformation calculations, nonparametric analysis was chosen. The categorical variables were described as frequencies with percentages and their associations with the CAD and non-CAD groups and with the prothrombin genotypes were compared using the χ^2^ test or the Fisher exact test, wherever appropriate. All the P values were 2-tailed, and the values of less than or equal to 0.05 were considered statistically significant. 

## Results

Among the 1460 patients undergoing coronary angiography, the mean age was 58.21±10.33 years and 65.3% were men. On coronary angiography, 19.6% of the patients had normal coronary arteries and 12.5% had minimal CAD. Single-vessel, double-vessel, and triple-vessel diseases were diagnosed in 20.3%, 18.2%, and 29.7% of the patients, respectively. The baseline characteristics of the studied patients with and without CAD are summarized in Table 1. Age and the proportion of men were higher in the CAD group than in the non-CAD controls. The prevalence rates of diabetes, family history of CAD, hyperlipidemia, and cigarette smoking were significantly higher in the CAD group, while there was no statistically significant difference in the prevalence of hypertension between the 2 groups.

As is seen in Table 2, in the univariable analyses, the allele and genotype distributions of the factor II G20210A were not statistically different between the patients with and without coronary stenosis. Given the rarity of the homozygous mutant genotype, the clumped AA+AG genotypes were compared with the GG genotype (Table 3). No statistically significant differences were found in the demographic data, the presence or absence of CAD risk factors, and the level of biochemical markers in terms of the factor II genotypes between the study groups. In addition, Table 3 shows that the median and the IQR for the Gensini score were not statistically significantly different between the GG and AA+AG genotypes (P=0.44). The association between the G20210A polymorphism and the severity of CAD with respect to the vessel score is also depicted in Table 3. As can be seen, there was no significant linear trend of higher numbers of diseased vessels (P= 0.765 for the Mantel–Haenszel test of linear trend) in the mutant genotypes (AA+GA) in comparison with the wild (GG) genotype.

**Table 1:** The study population characteristics based on the presence of coronary artery disease*

Variable	non-CAD (n=465)	CAD (n=995)	P value
Age (y)	56.00±10.27	59.23±10.18	<0.001
BMI (kg/m^2^)	28.20±4.70	27.60±4.29	0.037
Male sex	235 (50.5)	718 (72.2)	<0.001
Diabetes mellitus	97 (20.9)	315 (31.7)	<0.001
Hypertension	259 (50.7)	551 (50.9)	0.943
Cigarette smoking			<0.001
Current smoker	72 (15.5)	257 (25.8)	
Ex-smoker	70 (15.1)	200 (20.1)	
Nonsmoker	323 (69.5)	538 (54.1)	
Family history of CAD	81 (17.4)	224 (22.5)	0.026
Hyperlipidemia	266 (57.2)	694 (69.7)	<0.001
History of MI	36 (7.7)	417 (41.9)	<0.001
History of RF	2 (0.4)	20 (2.0)	0.021
Previous CABG	0	33 (3.3)	<0.001
Previous PCI	0	66 (6.6)	<0.001
Previous stroke	7 (1.5)	27 (2.7)	0.154
Fasting glucose (mg/dL)	107.40±33.39	120.44±49.33	<0.001
Creatinine (mg/dL)	1.13±0.43	1.22±0.57	<0.001

BMI, Body mass index; CABG, Coronary artery bypass graft; CAD, Coronary artery disease; MI, Myocardial infarction; PCI, Percutaneous coronary intervention; RF, Renal failure

* Data are presented as mean±SD or n (%).

**Table 2:** Allele and genotype distribution for the prothrombin G20210A gene variant in the patients with and without coronary artery disease

Factor II G20210A	non-CAD (n=465)	CAD (n=995)	P value
Gene variant			0.620
GG	443 (95.3)	944 (94.9)	
GA	20 (4.3)	42 (4.2)	
AA	2 (0.4)	9 (0.9)	
Allele frequency			0.886
G	906 (97.4)	1930 (97.0)	
A	24 (2.6)	60 (3.0)	

Data are presented as n (%).

CAD, Coronary artery disease

**Table 3:** Clinical, laboratory, and angiographic characteristics of the study population in association with prothrombin genotypes*

Variable	GG (n=1387)	AA+AG (n=73)	P value
Age (y)	58.13±10.28	59.63±10.93	0.166
Male sex	902 (65.0)	51 (69.9)	0.398
Diabetes mellitus	391 (28.2)	21 (28.8)	0.915
Hypertension	713 (51.4)	33 (45.2)	0.302
Cigarette smoking			0.856
Current smoker	314 (22.6)	15 (20.5)	
Ex-smoker	255 (18.4)	15 (20.5)	
Nonsmoker	818 (59.0)	43 (58.9)	
Family history of CAD	285 (20.5)	20 (27.4)	0.225
Hyperlipidemia	909 (65.5)	51 (69.9)	0.448
Fasting glucose (mg/dL)	116.18±45.30	118.41±45.02	0.582
Total cholesterol (mg/dL)	185.84±46.53	187.70±46.12	0.586
HDL cholesterol (mg/dL)	43.08±10.79	41.31±10.18	0.161
LDL cholesterol (mg/dL)	108.93±40.33	112.06±33.01	0.368
Triglyceride (mg/dL)	173.08± 98.27	179.40±107.90	0.907
Creatinine (mg/dL)	1.18±0.45	1.35±1.34	0.542
Gensini score	31.00 (3.000-72.00)	35.50 (4.00-91.50)	0.443
Number of diseased vessels			0.244
Absent**	274 (19.8)	12 (16.4)	
Minimal***	169 (12.2)	10 (13.7)	
Mild****	275 (19.8)	21 (28.8)	
Moderate*****	257 (18.5)	8 (11.0)	
Severe ******	412 (29.7)	22 (30.1)	

CAD, Coronary artery disease. HDL, High density lipoprotein. LDL, Low density lipoprotein

* Data are presented as mean±SD or n (%) except for the Gensini score which is presented as the median (IQR_25% –75%_).

** No coronary lesions

*** No coronary lesions with >50% luminal stenosis

**** Single-vessel disease with >50% luminal stenosis

***** Double-vessel disease with >50% luminal stenosis

****** Triple-vessel disease with >50% luminal stenosis

## Discussion

The presence of G to A transition in the 30 upstream untranslated region of the prothrombin gene at the nucleotide locus 20210 may play a regulatory role in gene expression.^   2 ^ In 1996, for the first time, Poort et al.^   2 ^ published the result of their research regarding a common genetic variation (G20210A) in the prothrombin gene that was associated with higher prothrombin levels and with an increased risk for venous thrombosis. The authors suggested that such a transition (located at or near the cleavage site of the mRNA precursor, to which poly A is added) stabilizes mRNA and allows the production of the corresponding protein to be increased. Several subsequent studies confirmed these initial findings.^16^^-^^19^  

The increased prothrombin levels associated with the G20210A variant may, at least theoretically, result in a higher risk for CAD since excessive thrombin generation has been described in men at high risk for fatal CAD.^   20 ^ In the present study, we evaluated the effects of the prothrombin gene polymorphism on CAD presence and severity in a relatively large Iranian sample. We found that there was no association between this polymorphism and either the presence or the severity of CAD among the study population. To our knowledge, this is the first study in an Iranian sample to examine the possible role of the prothrombin G20210A polymorphism in the severity of CAD as assessed with the Gensini score, which gives a more reliable estimate for the severity of coronary artery stenosis.^   12 ^

Prior evidence for the association between the prothrombin G20210A polymorphism and CAD is conflicting.^3^^, ^^21^^-^^24^  In some studies, being a carrier of the mutation was linked to a higher risk for myocardial infarction.^5^^, ^^25^^-^^27^  Segev et al.^   2 8^ showed that among young patients with myocardial infarction and a few traditional risk factors, the prevalence of the prothrombin gene G20210A variant was significantly increased. Several meta-analysis studies have proposed that the factor V Leiden (F5 rs6025) and the prothrombin gene G20210A (F2 rs1799963) polymorphisms are weak risk factors for CAD and stroke, particularly among younger patients and women. ^3^^, ^^29^^, ^^30^  In a recent comprehensive meta-analysis, the G20210A variant was associated with a significantly increased risk for CAD in Europeans but not in Americans or Asians.^   3 ^ Moreover, the only large prospective cohort study published to date failed to establish any link between the G20210A polymorphism and myocardial infarction or stroke.^   3 1^

One possible explanation for such a disparity may be the rarity of the mutant allele (A). The prevalence of the A allele was 2.9% among our relatively large study population (3.0% in the case group vs. 2.6% in the controls). The frequency of the A allele varies widely in different surveys with various sample sizes, in different geographical regions, and in different ethnic populations.^   3 ^ The prevalence of the A allele among Europeans ranges between 1.7 and 2%, and it is very rare in individuals of Asian and African descent.^   3 2^ In a study in western Iran, the prevalence of the A allele was reported to be 1.3% and 0% among patients with CAD and controls, respectively.^9^ The relatively small number of individuals with the A allele would lead to an insufficient statistical power for the detection of a modest association.

Another possible explanation for such a disparity is that a precise definition of the phenotype, which is crucial in designing genetic studies,^   33 ^ is difficult if not impossible, especially in subjects with clinically silent CAD. If coronary angiography is not performed, these patients may be regarded as controls, leading to an increased probability of null results. Thus, to avoid this potential bias in the present study, we defined cases and controls on the basis of objective angiographic documentation of the coronary artery status. Other explanations for the controversial results in different studies on the association between the prothrombin G20210A polymorphism and CAD may be the existence of variations in the frequency of other genetic and environmental risk factors in various populations and variations in selection criteria in different studies. 

A few studies have assessed the relationship between the prothrombin G20210A variant and the severity of CAD. 7^, ^^19^^, ^^34^  A pooled analysis demonstrated a higher prevalence of the GA genotype among patients with no or single-vessel disease than in those with multi-vessel disease (4.4% vs. 2.2%; relative risk: 2.0, 95% CI: 1.2–3.1).^   3 4^ In a case-control study, Russo et al.^19^ failed to show any relationship between the G20210A genetic variant and the severity of CAD in 660 consecutive patients referred for cardiovascular surgery. The case group (n=436) patients had angiographically documented severe multi-vessel CAD and were candidated for coronary artery bypass grafting, and the control group patients (n=224) had angiographically documented normal coronary arteries and were examined for reasons other than suspected CAD (mainly valvular disease). Elsewhere, in a study by Gundogdu et al.^   7 ^ on 268 unrelated subjects who were referred for coronary angiography due to suspected ischemic heart disease, this genetic variant was not significantly associated with CAD or its severity. All the abovementioned studies used the vessel score to determine the severity of CAD. Chiming in with our study, Gardemann et al.^        1 0^ estimated CAD severity by calculating the Gensini score and showed that the mean Gensini score was similar between FII G20210A genotypes in a population of 2210 male Caucasians who consecutively underwent coronary angiography. However, the investigators found that the carriers of the A allele had significantly higher Gensini scores than did the GG homozygotes in a subgroup of high-risk patients (i.e., smokers without acetylsalicylic acid treatment and patients with low apoAI/apoB ratios and high Lp(a) plasma levels). 

## Conclusion

Our data on a relatively large number of Iranian patients who underwent coronary angiography failed to confirm the hypothesis that the G20210A variant mutation is a significant determinant of CAD risk or its severity.

## Notes:

***This paper should be cited as: ***Pourgholi L, Goodarzynejad H, Ziaee S, Zare E, Jalali A, Boroumand M. Prothrombin Gene G20210A Variant in Angiographically Documented Patients with Coronary Artery Stenosis. J Teh Univ Heart Ctr 2019;14(4):150-155.
